# Selective Laser Melting and Electron Beam Melting of Ti6Al4V for Orthopedic Applications: A Comparative Study on the Applied Building Direction

**DOI:** 10.3390/ma13235584

**Published:** 2020-12-07

**Authors:** Paola Ginestra, Rosalba Monica Ferraro, Keren Zohar-Hauber, Andrea Abeni, Silvia Giliani, Elisabetta Ceretti

**Affiliations:** 1 Department of Mechanical and Industrial Engineering, University of Brescia, via Branze 38, 25123 Brescia, Italy; andrea.abeni@unibs.it (A.A.); elisabetta.ceretti@unibs.it (E.C.); 2 Institute of Molecular Medicine “Angelo Nocivelli”, Department of Molecular and Translational Medicine, University of Brescia, 25123 Brescia, Italy; rosalba.ferraro@unibs.it (R.M.F.); silvia.giliani@unibs.it (S.G.); 3 Metallurgical and Powders Technologies Lab, Institute of Metals, Technion City, Haifa 320003, Israel; kerenz@trdf.technion.ac.il

**Keywords:** powder bed fusion, selective laser melting, electron beam melting, building orientation, titanium, osseointegration

## Abstract

The 3D printing process offers several advantages to the medical industry by producing complex and bespoke devices that accurately reproduce customized patient geometries. Despite the recent developments that strongly enhanced the dominance of additive manufacturing (AM) techniques over conventional methods, processes need to be continually optimized and controlled to obtain implants that can fulfill all the requirements of the surgical procedure and the anatomical district of interest. The best outcomes of an implant derive from optimal compromise and balance between a good interaction with the surrounding tissue through cell attachment and reduced inflammatory response mainly caused by a weak interface with the native tissue or bacteria colonization of the implant surface. For these reasons, the chemical, morphological, and mechanical properties of a device need to be designed in order to assure the best performances considering the in vivo environment components. In particular, complex 3D geometries can be produced with high dimensional accuracy but inadequate surface properties due to the layer manufacturing process that always entails the use of post-processing techniques to improve the surface quality, increasing the lead times of the whole process despite the reduction of the supply chain. The goal of this work was to provide a comparison between Ti6Al4V samples fabricated by selective laser melting (SLM) and electron beam melting (EBM) with different building directions in relation to the building plate. The results highlighted the influence of the process technique on osteoblast attachment and mineralization compared with the building orientation that showed a limited effect in promoting a proper osseointegration over a long-term period.

## 1. Introduction

Longer life expectancy and increase in population will raise the volume of medical implantable devices, while the higher mobility demanded by younger patients may outlive such devices. This increase in demand is linked to the inherent complexity of the required implants that, paired with the differences between patients, makes their standardization difficult, originating the challenge to manufacture high-quality and different implantable devices [1,2]. Most of the current research on the design optimization of customized implants is focused on finding the optimal configuration to achieve the best performances in vivo [3,4,5,6]. Specifically, bespoke implants are produced to fit the patient’s anatomical districts of interest even considering complex implant sites [7,8]. One of the most used strategies for the fabrication of bespoke devices is topological optimization [9,10,11], which usually leads to the design of lattice-integrated parts, especially functionally graded lattices [12,13]. Moreover, an adequate choice in terms of biocompatibility and corrosion resistance is often related to the selection of the proper material for the majority of load-bearing prostheses that require certain mechanical properties to assure the long-term functionality. In contrast, all the metal implants used for orthopedic, maxillo-facial, dental, and cranial applications have to deal with short-term and long-term infections as well as with poor osseointegration and insufficient integration with the surrounding tissue [14,15,16,17].

To overcome these complications, the chemical and mechanical characteristics of these devices are being tuned accordingly. For example, several coatings have been exploited to reduce the biofilm formation [18,19,20] and increase the life expectancy of the implants [21,22,23], while tailorable mechanical responses can improve the formation of an optimal interaction with the physiological tissue [24,25,26], reducing the stress-shielding effect at the interface that is responsible for most of the failures of these devices. Among these perspectives, the surface composition and morphology are crucial parameters that influence the primary response of the body to an implant depending on the anatomical region [27]. In particular, the roughness of the surface and the absence of contaminants and other impurities are responsible for achieving the desired cell recognition and the decrease of the long-term infection rates (the short-term infections being mostly related to the surgery procedure) [28,29]. Typically, the surface of a metal implant is treated with passivation, vibratory finishing, and grinding techniques in order to obtain a mirror polishing effect at the end of the post-processing chain. A totally polished surface can indeed drastically reduce the bacterial adhesion and proliferation but, at the same time, hinder the cell attachment and colonization of the implant. One of the disadvantages of the additive manufacturing (AM) technologies over the conventional techniques is the requirement of post-processing operations due to the inadequate dimensional accuracy and surface finishing that result from a layer-by-layer deposition of material [30,31,32,33]. Moreover, the advantages of the lead time reduction offered by 3D printing are often affected by the time-consuming techniques, including manual operations for curved or complex geometries, required to modify the surface quality of as-build samples.

The additive manufacturing process of metals has been analyzed to identify and optimize the relation between the process parameters, the internal metallurgical defects, and the residual thermal stresses that have a significant impact especially on the mechanical properties of the parts [34,35,36,37,38]. The relation between the printing parameters and the resulting surface quality, especially for convoluted structures, is still under investigation [39,40]. So far, the powder bed fusion (PBF) techniques, Selective Laser Melting (SLM) and electron beam melting (EBM), are used the most for the fabrication of customized metal implants [41,42,43]. In recent years, many researchers have devoted their studies to 3D printing of Ti6Al4V titanium alloy for biomedical applications [44,45,46,47]. Recently, a few research papers have reported comparative studies between the two techniques to highlight the differences mostly in terms of corrosion resistance and fatigue life of SLM and EBM titanium samples [48,49]. Precisely, the surface defects of the SLM and EBM samples, which remained after Hot Isostatic Pressuring (HIPing)and machining, have been demonstrated as crucial for the stress concentration during fatigue tests, being the dominant mechanisms for lifetimes lower than 107 cycles [50,51]. Moreover, the surface irregularities of the EBM samples were more significant than those found in the SLM samples [52]. Furthermore, the surface state of SLM and EBM samples can influence the corrosion rates and thus the in vivo applications [53]. Nevertheless, a possible control of the surface properties depending on the process optimization has yet to be fully described. One of the most recently studied parameters to analyze the outcomes of additive technologies on the final properties of metal samples is the building strategy [54,55,56,57,58,59]. Differently from the other parameters of the printing process that are often previously optimized and set according to the material, the equipment, and the selected application, the building direction is an investigable, editable, and optimizable constraint that has been demonstrated to affect different features of as-built metal specimens. Several studies focus on the effects of the building orientation on the mechanical properties of 3D printed metal alloys, especially titanium-based blends. Specifically, the corrosion resistance, microstructure anisotropy, and tensile behavior dependence on the building direction have been reported for EBM fabricated parts [54,55,56].

Furthermore, SLM samples have been produced with different building orientations to determine how the tensile, fatigue, and general anisotropies of their mechanical properties can be affected by this parameter [57,58,59]. This paper aimed to introduce a critical comparison between Ti6Al4V samples fabricated by SLM and EBM at different building angles. In particular, four building angles were used, i.e., 0°, 15°, 30°, and 45°, to fabricate cubical samples and characterize the final surface morphology, contact angle (CA), cross-section, and roughness. The objective of this work was to highlight the possibility of tuning the surface properties of metal additively manufactured samples with a proper design of the building strategy for the production of orthopedic implants. For this reason, biological tests were carried out to investigate the relation between the surface characteristics of the specimens and osteoblast adhesion, proliferation, and mineralization mechanisms.

## 2. Materials and Methods

### 2.1. Selective Laser Melting and Electron Beam Melting of Ti6Al4V Samples

**Figure 1:**
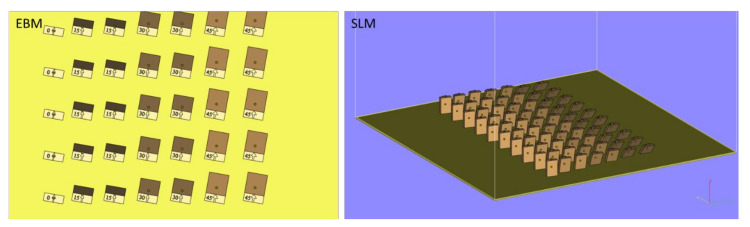
Building orientation and positioning of the Electron Beam Melting (EBM) and Selective Laser Melting (SLM) samples on the printer plate.

Four building angles to produce the implant models have were considered. The angles chosen for the laser building strategy were 0°, 15°, 30°, and 45° in relation to the building plate for both the EBM and SLM processes taken into account (Figure 1).

The angles were varied to mimic a design feature that can be implemented on an implant, e.g., to promote osseointegration or functionalize the surface without a specific post-processing step. These designs were considered to assess whether the printing process could affect the surface properties and, in turn, cellular adhesion properties of samples additively manufactured using EBM and SLM. Cubical samples (10 × 10 × 10 mm^3^) were fabricated from Ti6Al4V (Ti64) inert-gas atomized powder (EOS Titanium Ti6Al4V and ARCAM Ti64Al4V powders) using an EBM SYSTEMS MODEL A2 (ARCAM, Gothenburg, Sweden) and an EOS M290 (EOS, Krailling, Germany) for the EBM and SLM samples, respectively. Table 1 reports the relevant properties of the used Ti6Al4V powders according to the required datasheets according to ASTM B214, B215, B212, F2924 and F1472 [60,61,62].

The EBM system operates a scanning strategy with an alternating angle between the layers of 90°. The parameters used to fabricate the EBM samples were 1250 W of beam power, 80 μm of beam focus diameter, 4530 mm/s scanning speed, hatch spacing of 100 μm, and slice thickness of 50 μm. Support structures were built between the substrate base and each sample to provide stability during the build using the following parameters: 800 W of beam power and 1400 mm/s scanning speed. The SLM scanning strategy was set with an alternating angle between layers of 67°. The parameters used to produce the SLM samples were 340 W of laser power, 70 μm of laser focus diameter, 1250 mm/s scanning speed, hatch spacing of 40 μm, and slice thickness of 30 μm. Support structures were built to provide stability with a laser power of 100 W and 600 mm/s scanning speed. The manufactures were conducted in a controlled atmosphere with argon gas to minimize oxygen pick-up to <0.1%. The supports were removed and the samples were cleaned using a standard cleaning methodology (sonication in acetone and isopropanol) and left to dry.

### 2.2. Characterization of Additively Manufactured Parts

#### 2.2.1. Analysis of Surface Topography Using Scanning Electron Microscopy

Ti64Al4V EBM and SLM specimens were observed and topographical imaging was performed using an Inspect™ environmental SEM (FEI, Tokyo, Japan) operating at 20 kV. The samples were attached to aluminum stubs with double-adhesive carbon tabs. The samples for cross-section evaluation were embedded in cold resin epoxy and then polished. Subsequently, the surface profiles of the cross-section of Ti6Al4V EBM and SLM specimens were examined.

#### 2.2.2. Three-Dimensional Surface Reconstruction and Roughness Analysis Using Optical Microscopy

Optical imaging of the as-fabricated EBM and SLM parts at different building angles was performed using a Wyko NT1100 3D Optical profilometer (Veeco, Plainview, NY, USA) at 2.5x magnification. A scanning size of 0.92 × 1.2 mm^2^ was selected at the central point of the surface with a sampling of 1.65 µm. The scanning was carried out between the maximum and minimum focusing points of the z height of the EBM and SLM sample surfaces. The roughness parameters (*Ra*, *Rq*) were assessed and analyzed using the Vision Veeco Module (Veeco, Plainview, NY, USA).

#### 2.2.3. Surface Wettability

Contact angle measurements were obtained using the Attension Theta Lite optical tensiometer (KSV NIMA Biolin Scientific, Västra Frölunda, Sweden). The EBM and SLM samples were placed on the bottom flat surface and a droplet of deionized water (5–10 μL) was pipetted into the center of the top surface (considering the different building angles) at room temperature. The images of the contact angle were captured following the horizontal plane of the droplet for 30 s after application. The OneAttension software converts the images to 8-bit greyscale to obtain the inner angle between the surface and air/water interface.

Single-factor analysis of variance (ANOVA) tests were conducted to verify any significance within samples (i.e., between samples of the same design and technique). This was used as an assessment of reproducibility. A two-way ANOVA with factors as design at four levels (0°, 15°, 30°, 45°) and powder bed technique (at two levels, SLM and EBM) with post-hoc Tukey tests was conducted to determine if both the building angle and the powder bed fusion technique significantly altered the contact angle and the roughness of the samples. Statistical tests were performed at α = 0.05 using Minitab 18^®^ (MINITAB, Coventry, UK).

### 2.3. In Vitro Adhesion, Proliferation, and Mineralization of MC3T3 Preosteoblast Cells

#### 2.3.1. Cell Culture and Seeding

MC3T3-E1 Subclone 4 cells (BS CL 181, IZSLER, Brescia, Italy) from passage 36 were cultured in a proliferation medium composed of Alpha Minimum Essential Media (α-MEM) with sodium bicarbonate, ribonucleosides, and deoxyribonucleosides (BioConcept, Allschwil, Switzerland) supplemented with 10% FCS (Euroclone, Milan, Italy) and 100 units/mL pen/strep (Euroclone, Milan, Italy) in a humidified incubator set at 37°C and 5% CO_2_. Prior to in vitro experiments, samples were washed in phosphate-buffered saline (PBS) (Euroclone, Milan, Italy), autoclaved, and put into 12-well plates with a sterile tweezer. For all assays, a culture of MC3T3-E1 osteoblasts in 2D on plastic well plates was conducted in parallel as positive control. MC3T3-E1 cells were detached using trypsin/EDTA (0.25% *w*/*v* trypsin/0.02% EDTA) from a plate culture at maximum of 70% of confluence, diluted in a little volume of medium and seeded (5 × 10^4^ cells/cm^2^) onto the top surface of titanium scaffolds and tissue cultured. Cells were allowed to adhere for 1 h before adding complete growth media to each well to cover the sample surfaces. Cells were maintained at 37°C in a saturated humidity atmosphere containing 95% air and 5% CO_2_. The mineralization medium was prepared by adding 50 µg·mL^−1^ ascorbic acid (Sigma-Aldrich), 10 mM β-glycerophosphate (Sigma-Aldrich, St. Louis, MO, USA), and 100 nM dexamethasone to the cell culture medium. The mineralization medium was changed every 3 days.

#### 2.3.2. Cell Viability Assay

A CellTiter -Glo 3D Cell Viability (rATP) assay kit (Promega, Madison, WI, USA) was used to determine the number of viable cells in 3D titanium cultures. This test is based on quantitation of the adenosintriphosphat (ATP) present, which is directly proportional to the number of metabolically active cells. The assay was performed according to supplier protocols after 24, 48, and 96 h of culture on triplicate samples. Briefly, a volume of CellTiter-Glo 3D Reagent equal to the volume of cell culture medium present in each well was added to induce cell lysis. After incubation at room temperature (RT) for 25 min to stabilize the luminescent signal, the total volume was transferred into a 96-well opaque-walled multiwell plate and the luminescence was recorded using a Microplate reader Infinite 200 (Tecan, Männedorf, Switzerland). An ATP standard curve was generated to compare luminescence of samples to luminescence of standard and determine the amount of ATP detected in the samples. At each time point, unpaired t-test was conducted to determine if the different surface treatments significantly altered the metabolic activity in comparison to the control samples cultured on plastic well plates.

#### 2.3.3. Immunofluorescence Staining

To study cell adhesion, MC3T3-E1 cells were seeded (5 × 10^4^ cells/cm^2^) on the surface of samples from each group and incubated at 37°C in 5% CO_2_. After 7 days of culture, cells on substrates were fixed and permeabilized using a Fir & Perm Reagent kit (Nordic MUbio, Susteren, The Netherlands). Then, blocking solution iBindTM Buffer (Invitrogen, Carlsbad, CA, USA) was applied for 45 min, and stained for 30 min with phalloidin (Thermo Fisher Scientific, Waltham, MA, USA) that marks the cytoskeletal component of the cells. Cell nuclei were then counterstained with Hoechst 33342 (Thermo Fisher Scientific) for 5 min to highlight the nuclei of living cells. Cells were observed with an inverted fluorescence microscope (Olympus IX70, Tokyo, Japan), and images were analyzed with Image-Pro Plus software v7.0 (Media Cybernetics, Rockville, MD, USA).

#### 2.3.4. Alizarin Red Staining

Alizarin Red (AR) is used in histology to stain calcium deposits in tissues. Cells were washed twice with phosphate-buffered saline, fixed with reagent B from the Fix & Perm Reagent kit (Nordic MUbio) for 15 min, washed twice with distilled water, and stained with 40 mM Alizarin Red (Sigma-Aldrich) at RT with gentle shaking for 10 min, then washed three times with distilled water and observed.

#### 2.3.5. RNA Extraction and Quantitative Real-Time PCR (qPCR)

In order to evaluate the mineralization status of each culture, after 15 and 30 days in culture, total RNA was isolated from the cells adhered to the scaffold using a NucleoSpin^®^ RNA II kit (Macherey-Nagel, Düren, Germany) and quantified using a spectrofluorometer (Infinite 200, Tecan). RNAs were retro-transcribed using the ImPromIITM Reverse Transcription System (Promega), following the manufacturing protocol. A turbo DNAse treatment (Thermo Fisher Scientific) was applied to remove all the residual genomic DNA, then qPCR was performed using iTaq Syber Green (Promega)-based assays following protocol: 3 min at 95 °C for polymerase activation followed by 40 cycles of 10 s denaturation at 95 °C, 20 s annealing at 60 °C, and 1 s extension at 72 °C. MC3T3-E1 mineralization-related primers (Table 2) were used to check the gene expression at the mRNA level, including bone sialoprotein (*Bsp*), osteocalcin (*Ocn*), osteopontin (*Opn*), and matrix extracellular phosphoglycoprotein (*Mepe*). The specificity of the primers was tested separately before the reaction and the negative control consisted of RNase-free water. Assays were performed on the CFX96 C1000 TouchTM Real-Time PCR Detection System, and analyzed with CFX manager software v.3.1 (BioRad, Hercules, CA, USA). The relative quantification of target genes was calculated by the 2^−ΔΔCt^ method, using *Gapdh* as housekeeping gene for normalization of the data.

## 3. Results

### 3.1. Surface Topography of the EBM and SLM Samples

**Figure 2:**
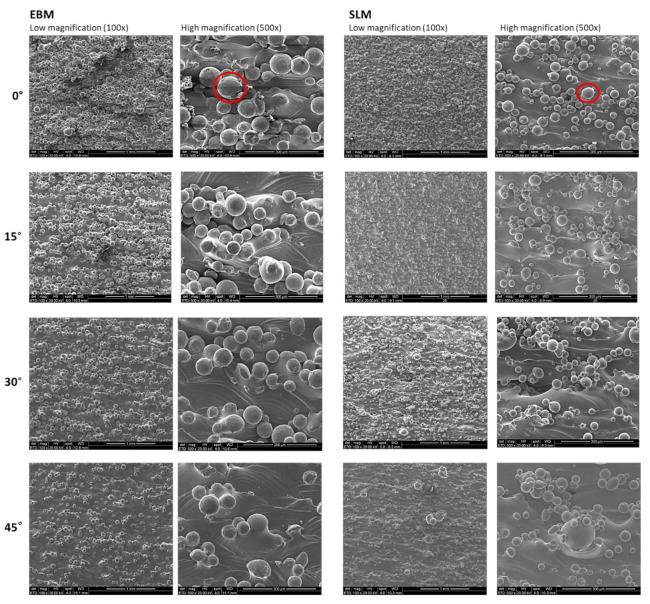
Micrographs illustrating the topography of the as-built surfaces of Ti6Al4V parts manufactured by EBM and SLM at 0°, 15°, 30°, and 45°. Red cycles highlight the partially melted particles.

Scanning electron micrographs reveal the presence of a building strategy for each sample and highlight the differences between the scanned surfaces depending on the technology used to manufacture parts (Figure 2). Spherical partially melted powder particles are observed on all the surfaces of the EBM and SLM samples, but fewer particles are visible as the building angle is increased (Figure 2). In particular, the EBM samples exhibit notably different surface topographies compared to SLM surfaces due to the presence of bigger particles and in greater amounts.

**Figure 3:**
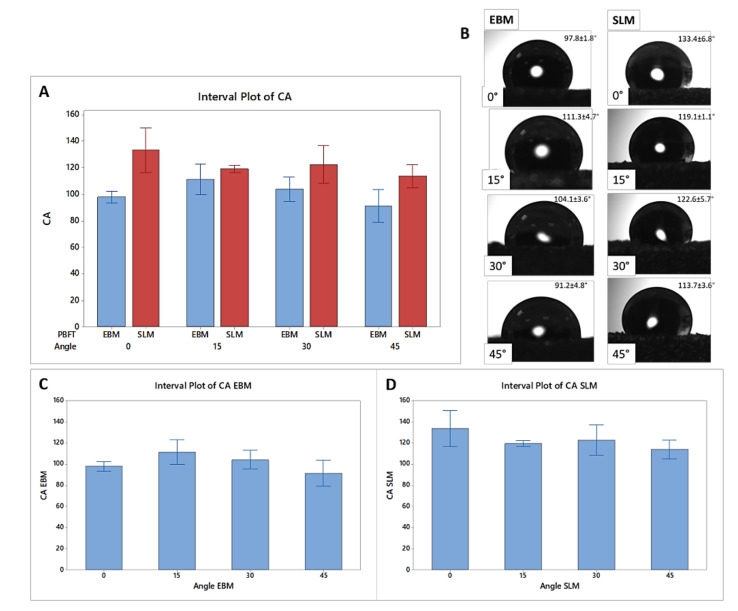
Contact angle (CA) measurements for the EBM and SLM samples: (**A**) comparison between average data within each group and (**B**) images of the water droplets on the top surfaces at different building angles; contact angle measurement for the (**C**) EBM individual samples and (**D**) SLM individual samples.

In particular, the particles on the SLM surfaces have distinguishable dimensions in relation to the uniformly distributed particles on the EBM samples. In contrast, the scanning strategy of the EBM samples seems to lead to a less homogeneous surface due to the presence of agglomerates of particles at lower building angles and to the alternance of smooth scanning paths and particles lines at higher building angles. Furthermore, the presence of spherical particles seems to decrease as the building angle of EBM samples increases, while the difference between particles is less visible on the SLM surfaces built at different angles. Notably, the contact angle (CA) of the SLM surfaces is higher than that of the EBM at all the building angles, indicating that the PBF technology has a certain influence on the hydrophilic properties of the metal samples (Figure 3).

However, it is interesting to observe that, in this work, changing the building angle is not demonstrated to have a significant effect on the hydrophobicity of the surfaces for both the SLM and EBM techniques. The one-way ANOVA test performed on the contact angle measurements of the SLM samples reveals the existence of two significant groups of data, as shown in Table 3.

The same test performed on the contact angle measurements of the EBM samples results in the identification of three significant groups of data, as reported in Table 4.

**Figure 4:**
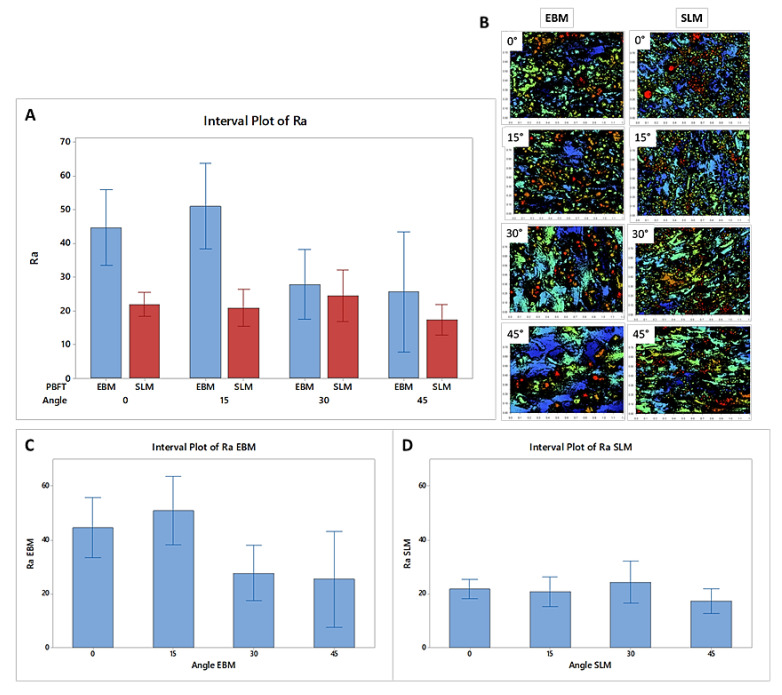
Roughness measurements (*Ra*) for the EBM and SLM samples: (**A**) comparison between average data within each group and (**B**) images of the surface mapping at different building angles; roughness measurement for the (**C**) EBM individual samples and (**D**) SLM individual samples.

For each sample, three measurements of Ra were obtained on the same sampling length (1.65 µm) chosen on the basis of the expected roughness of the examined surfaces. A significant difference between measurements of samples produced using EBM is observed at all the applied building angles, whereas a lower variance is reported for the roughness of the SLM samples, suggesting that *Ra* is reproducible within these groups (Figure 4).

On the contrary, the Powder Bed Fusion technology significantly affects *Ra* (*p* < 0.01), whereas the building angles used for the production of the SLM samples do not affect the recorded Ra values in the same way (*p* < 0.05). SLM is shown to reduce the average Ra values for all the samples produced at different building angles (21.9 ± 1.4, 20.8 ± 2.2, 24.4 ± 3.1, and 17.4 ± 1.8 for 0°, 15°, 30°, and 45°, respectively) when compared to EBM equivalents (44.7 ± 4.5, 51.0 ± 5.1, 27.8 ± 4.1, and 25.6 ± 7.2, respectively). As shown in Table 5, the SLM technique leads to a limited variation of the Ra and the one-way ANOVA test identifies two groups of data in which a poor reduction of the Ra is observed when the building angle is increased (Table 5).

Table 6 shows that the EBM technology exhibits a significant reduction of the Ra depending on the increase in the building angle (*p* < 0.01). Two groups are shown in this case, where the variation of the Ra between the samples printed at the lowest and highest building angles is observed (Table 6).

**Figure 5:**
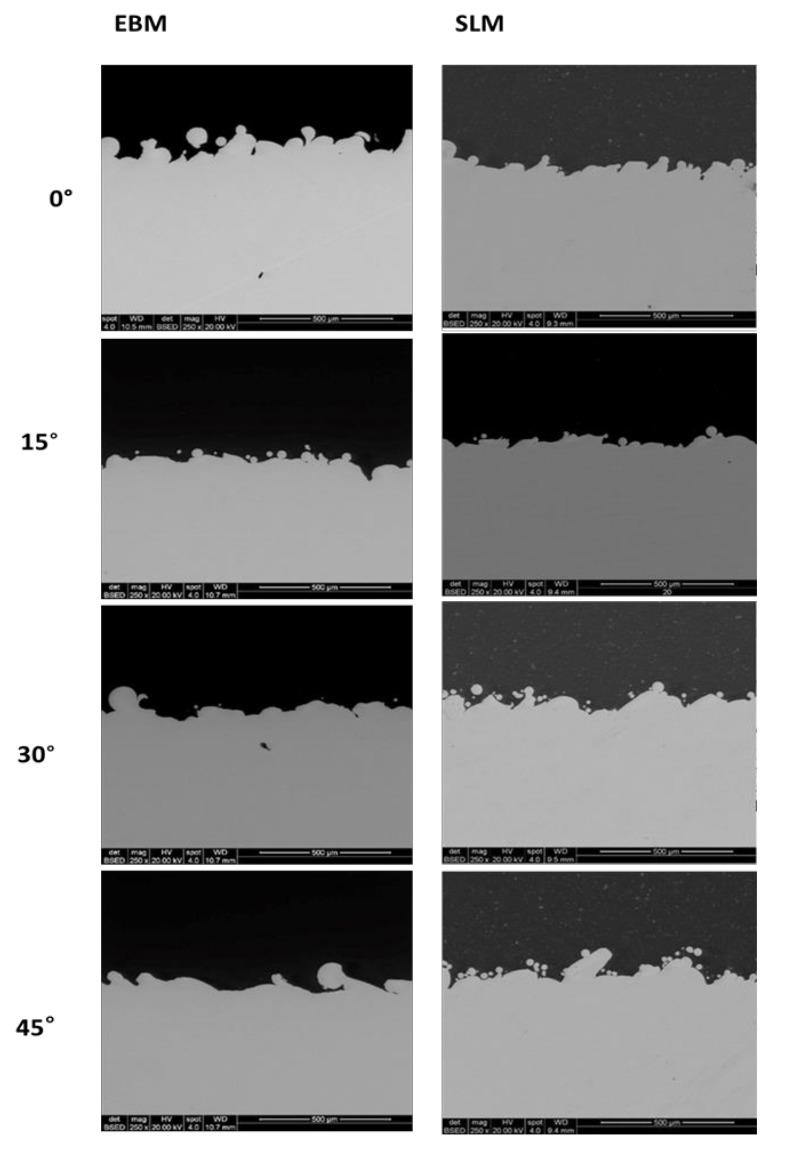
Micrographs (250x, scale bar is 500 µm) illustrating the surface topography of the cross-sections of the EBM and SLM samples built at different building angles.

The SEM metallographic cross-section samples show the surface profile of the top surfaces of the different printed samples (Figure 5).

It is observed that the profiles along the cross-sections of the SLM samples at different printing angles show a similar trend. The EBM samples show a flattening of some sections as the angle increases. The surface structures are formed as a result of partial melting of the powder on the top layer. The surface quality is highly dependent on powder size and, therefore, in this case, the SLM samples show a higher surface quality.

### 3.2. Viability and In Vitro Adhesion of MC3T3-E1 Cells on Titanium Scaffolds

A good scaffold for tissue integration must be biocompatible and sustain adhesion and proliferation of the surrounding host cells.

The biocompatibility of a material refers to its ability to provide appropriate support to cellular activity, including the induction of molecular and mechanical processes, which would optimize tissue regeneration, without producing any undesirable response in the host, either at a local or systemic level. The modulation of cell attachment, differentiation, and proliferation is instead more correlated to substrate characteristics, such as rigidity, thickness, roughness, and hydrophobicity, that can affect the cellular adhesiveness and migration. In order to test the biocompatibility and the adherent capacitance on the different angles of fabrication of the EBM and SLM scaffold surfaces, an attachment-dependent mouse cell line was cultured on the surface, namely MC3T3-E1. This cell line is obtained from the cranium of the mouse (*Mus musculus*) and is an example of a unipotent cell that can differentiate into an osteoblast with a fibroblast morphology [63].

**Figure 6:**
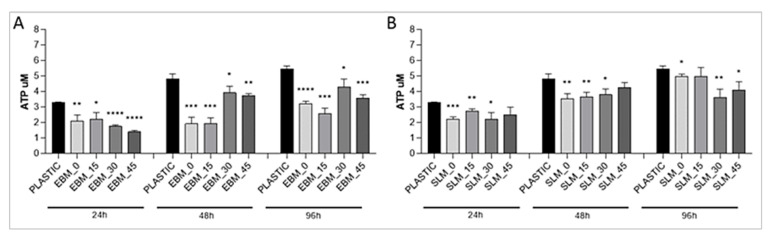
Measurement of the number of viable cells in culture with a luminescent signal proportional to the amount of ATP present. ATP (μm) concentrations of MC3T3-E1 cells cultured on EBM (**A**) and SLM (**B**) titanium scaffolds and on a plastic well plate (as control), estimated at different time points (24 h, 48 h and 96 h). Error bars represent SD derived from three different experiments (*n* = 3). Significant differences were analyzed between the positive control (cells on plastic dish) and each scaffold using unpaired *t*-test. A value of *p* < 0.05 was considered statistically significant (* *p* < 0.05, ** *p* < 0.01, *** *p* < 0.001, **** *p* < 0.0001).

To study cell viability, MC3T3-E1 cells were seeded onto EBM and SLM scaffolds and analyzed after 24, 48, and 96 h in culture. The samples to be evaluated were moved into a new empty culture plate prior to performing the viability rATP assay. This test allows to determine the number of viable cells by measuring the ATP concentration, which is directly proportional to the number of metabolically active cells. Figure 6 shows ATP (μM) concentrations (time points 24, 48, and 96 h) of MC3T3-E1 cultured onto plastic, as internal control, and onto EBM and SLM scaffolds. Specifically, a positive growth trend of proliferation is observed for MC3T3-E1 on the plastic dish, whereas a variable behavior is detected for cells grown on the titanium substrates. Cells on the EBM samples show a robust significant reduction in metabolic activity, more evident after 24 h from attachment, in comparison to the control on plastic (*p*-value < 0.0001). By contrast, the cellular behavior on the SLM samples shows a growth pattern more similar to the plastic control, manifesting some significant differences only in the lower angles of fabrication (*p* < 0.05).

**Figure 7:**
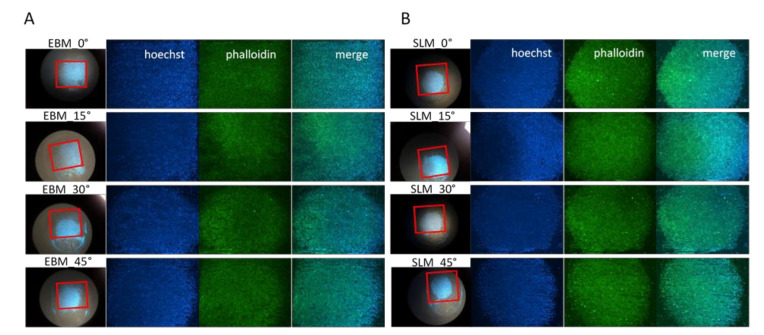
Fluorescence microscopy images of MC3T3-E1 cells after 7 days in culture on EBM (**A**) and SLM (**B**) titanium scaffolds (phalloidin in green, nuclei in blue, 2x), with a detailed photograph of the entire scaffolds delimited by a red square to indicate the area covered by the cells.

To investigate the cellular attachment, MC3T3-E1 cells were observed to adhere to all titanium surfaces after 7 days of culture through immunofluorescence assay. Cells were stained with phalloidin that is a specific marker for the cytoskeletal component, while nuclei were counterstained with Hoechst 33342 that highlights living cells. Fluorescence microscopy images in Figure 7 illustrate how both EBM and SLM scaffolds sustain the cellular adhesion, but in a different manner. Notably, MC3T3-E1 cells on the titanium EBM surface are seen to spread over all the surface, especially in the scaffolds with a lower angle of fabrication where some cells are stained also on the oblique sides at the edge of the support (Figure 7A). In contrast, MC3T3-E1 cells on the SLM substrates are detected to be clustered together, as can be clearly seen from the immunofluorescence images that reproduce the drop’s edge of the cell suspension plated on each support (Figure 7B).

### 3.3. Osteogenic Differentiation of MC3T3-E1 Cells on Titanium Scaffolds

Osteogenic differentiation of osteoblast-like cells seeded on scaffolds is of paramount importance for successful bone regeneration. The preosteoblastic MC3T3-E1 cell is a useful model to study osteogenesis in vitro because its temporal pattern of osteoblast differentiation is similar to in vivo bone formation. During the initial proliferative phase, cells go through a massive DNA synthesis and cell division, resulting in a rapid increase in cell number until confluence. Then, proliferation is arrested, and there is an increase in the sequential expression of mature osteoblastic characteristics including alkaline phosphatase (ALP) expression, conversion of procollagen to collagen, and the deposition of extracellular matrix containing calcium and additive proteins such as bone sialoprotein (BSP), osteopontin (OPN), matrix extracellular phosphoglycoprotein (MEPE), and, as a late-stage marker, osteocalcin (OCN) on the substrate, which is subsequently mineralized [64].

**Figure 8:**
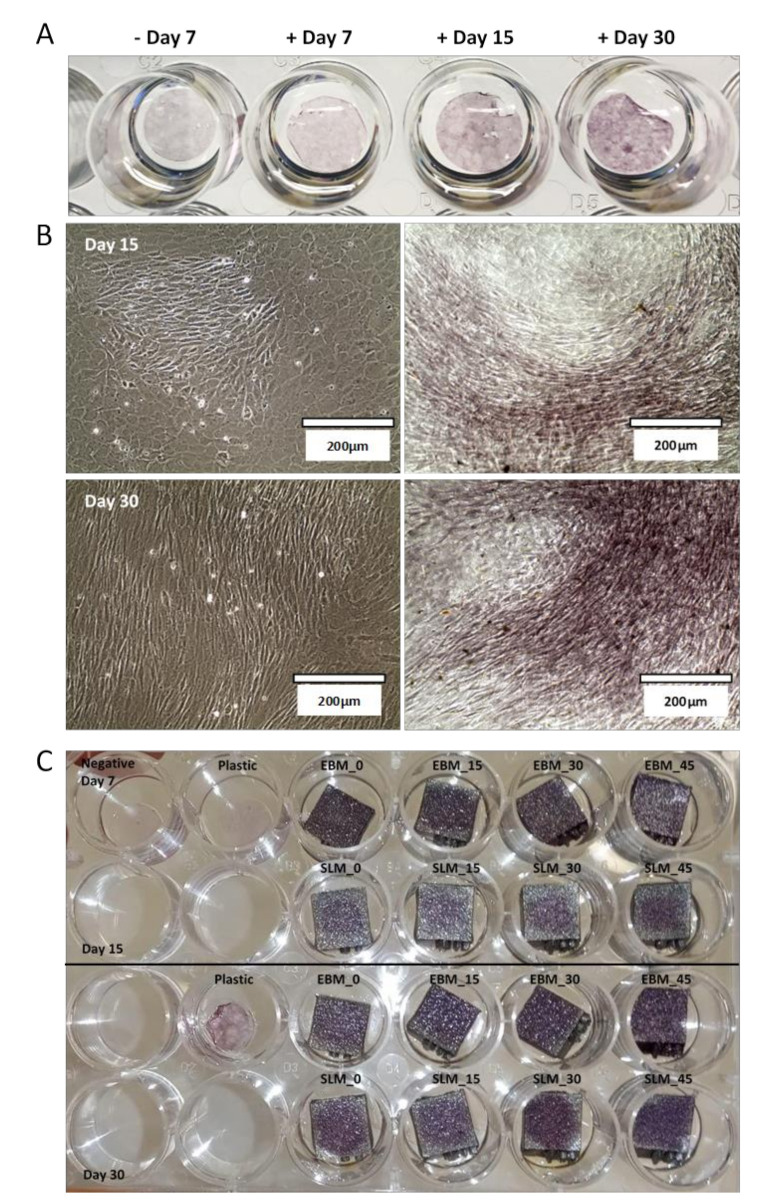
Alizarin Red staining of MC3T3-E1 cells cultured on plastic well plates in basal medium after 7 days (negative control; Day 7 refers to cell growth in basal medium) and in mineralization medium after 7, 15, and 30 days (**A**). Optical microscopic images in bright-field on the left and after ARS staining on the right of MC3T3-E1 cells cultured on a plastic dish in mineralization medium after 15 and 30 days (**B**). ARS staining of MC3T3-E1 cells cultured on EBM and SLM titanium scaffolds after 15 and 30 days in culture and on plastic well plate as positive and negative controls (**C**).

In order to verify that mineralization occurred, the protocol of differentiation was first corroborated by culturing MC3T3-E1 on a plastic well plate in the mineralization medium for 30 days, and performing Alizarin Red S (ARS) staining after 7, 15, and 30 days. In parallel, cells grown in basal medium for 7 days were stained as negative control. ARS is a dye that binds to calcium deposits of mineralization found in the mineralized extracellular matrix produced by osteoblasts, which provides a systematic qualitative tool to examine the osteoblastic differentiation in terms of time and substrate-dependent properties. MC3T3-E1 cells grown in the mineralized medium show increasing levels of orange–red staining of calcium nodules with the passing of the days in culture, proving the enhanced effects of mineralization supplements in the osteogenic medium. No significant positive ARS staining was detected for cells grown in basal medium for 7 days used as negative control (Figure 8A; Day 7 refers to cell growth in basal medium). Moreover, macroscopy pictures in bright-field and after ARS staining show how cells that start to mineralize tend to modify their morphology, becoming more elongated, and are promoted to cover the surface in a tight and ordinary manner (Figure 8B). ARS staining was also used to evaluate calcium-rich deposits by cells in culture in mineralization medium on titanium scaffolds at two time points (after 15 and 30 days) and in basal medium after 7 days as negative control. The result is illustrated in Figure 8C. Specifically, ARS staining shows the time course of increasing calcium mineralization on both EBM and SLM titanium scaffolds and in parallel on positive and negative controls on the plastic well. The general pattern presented is similar to the one that occurred in the immunofluorescence experiment used to assess the cellular adhesion, that is, on EBM scaffolds the calcium deposits are distributed on all the surface, whereas on SLM scaffolds they are mainly condensed in the middle. The calcium stain intensity is much higher after 30 days of mineralization, with no particular differences between the two scaffold typologies.

**Figure 9:**
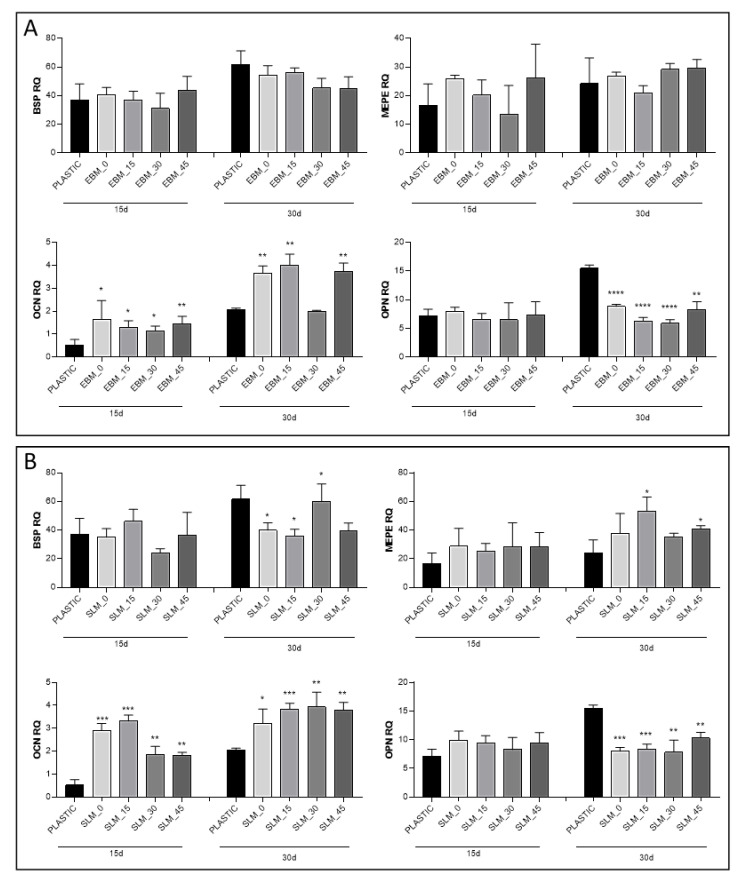
Osteogenic gene expressions of MC3T3-E1 cells cultured in mineralization medium after 15 and 30 days on EBM (**A**) and SLM (**B**) titanium scaffolds. The graph shows the relative quantification (RQ) of *Bsp, Mepe, Ocn*, and *Opn* genes normalized with *Gapdh* and calculated using as calibrator (RQ calibrator = 1) the constitutive gene expression of MC3T3-E1 cultured in basal medium. Error bars represent SD based on triplicates derived from three different experiments (*n* = 3). Significant differences were analyzed using *t*-test. A value of *p* < 0.05 was considered statistically significant. (* *p* < 0.05, ** *p* < 0.01, *** *p* < 0.001, **** *p* < 0.0001).

Finally, to compare the effect of growth on titanium scaffolds on the osteogenic capacity of MC3T3-E1 cells in a quantitative manner, the expression of genes involved in osteogenesis (*Bsp, Mepe, Ocn*, and *Opn*) was further determined performing quantitative real-time (RT) PCR (qPCR). Gene expression was analyzed after 15 and 30 days of mineralization. The relative quantification of target genes was calculated by the 2^−ΔΔCt^ method, using *Gapdh* as housekeeping gene for sample normalizations. The constitutive gene expression of the same markers was analyzed in MC3T3-E1 cells cultured in basal medium without mineralization and used as calibrator. The qPCR results of both control and scaffold groups are presented in Figure 9A, B for the EBM and SLM substrates, respectively. In the control group (cells grown on the plastic well plate), the expression of all detected genes is enhanced from Day 15 to Day 30. In the scaffold groups, the gene expression pattern is similar to the control one for *Bsp* and *Mepe* markers, while revealing an opposite scenario for the residual genes. Specifically, the *Ocn* gene expression on both scaffold groups shows a significant increase with respect to the control group on Day 15 and Day 30 (*p*-value < 0.05). On the contrary, the *Opn* gene expression on the scaffold groups is significantly reduced in comparison to the control group on Day 30 (*p*-value < 0.0001). The differences between *Ocn* and *Opn* gene expressions as compared to the control group are more evident in cells grown on SLM scaffolds. The results indicate that both EBM and SLM titanium scaffolds sustain the osteogenic differentiation of MC3T3-E1 cells, with statistical differences more evident in SLM substrates, in comparison with EBM. Moreover, the growth on a titanium scaffold seems to accelerate the mineralization in comparison to the growth on a plastic dish. This is confirmed by the statistically significant increase in the late-stage marker *Ocn* gene expression already visible at Day 15 in the scaffold group with respect to the cells grown on a plastic dish and by the statistically significant reduction of *Opn* gene expression in the scaffold group on Day 30 in comparison to control.

## 4. Discussion

The manufacturing processes presented in this work are characterized by high-temperature gradients, complex thermal properties typical of the materials processed with powder bed fusion techniques and critical optimization requirements. For these reasons, it is hard to guarantee the repeatability of the produced parts and the consistency of the material properties that would be necessary to allow the metal AM processes to be adopted widely by industry. Different techniques of PBF can be used for the fabrication of devices with distinctive properties due to the alteration of the microstructure caused by the powder fusion that can be suitable for a specific application [65]. Several works demonstrated how the building angle of a melting process, laser- or electron beam-based, can substantially change the surface, mechanical, and chemical properties of a metal part [66,67,68]. Here, the influence of both the powder bed fusion technique and the applied building angles on the properties of manufactured Ti6Al4V samples is demonstrated. The selective laser melting and electron beam melting techniques were compared as the most used for the production of AM metal implants. Four building angles (0°, 15°, 30° and 45°) were also compared to identify the relation between the manufacturing process and the process designs. When comparing micrographs of SLM and EBM samples, the surfaces appear macroscopically similar and both exhibit the presence of partially melted particles observed across the entire surface area of the top faces. In particular, the EBM surfaces are characterized by agglomerates of particles especially at lower building angles, whereas the SLM surfaces show the presence of uniformly distributed particles at all building angles (Figure 2).

The similarities may explain why no significant change in the viability values of MC3T3-E1 cells is reported between the SLM and EBM samples, while the differences may have caused the different spreading of cells on the SLM and EBM surfaces. In particular, the higher hydrophobicity of the SLM samples demonstrated at all the building angles when compared to the EBM equivalents explains the localized spreading of the cells on the SLM surfaces (Figure 3). Generally, comparable levels of cellular metabolic activity are measured for both SLM and EBM samples, but a certain difference between the plastic control and the metabolic activity of the cells at lower (0°, 15°) and higher (30°, 45°) building angles is observed for the EBM samples (Figure 6). This trend may be explained by the identification of the same groups of EBM samples when evaluating the roughness values statistically. Notably, the viability of the cells on the EBM samples is generally lower than that of the SLM samples, especially on the surfaces with a higher roughness, probably due to the greater variation of the data observed for the EBM samples, indicating the random presence of agglomerates of particles that may eventually facilitate tissue ingrowth in certain areas but impede the uniform spreading of cells due to the large angle between the spherical particles and the bulk surface as already reported [69]. In fact, the micrograph images and roughness measurements reveal that EBM surfaces are qualitatively and quantitatively not spatially consistent across the analyzed surface (Figure 4 and Figure 5). It has been observed that, during the contact angle measurements, the water droplet immediately flattened on the EBM surfaces, suggesting that it penetrated the samples. Moreover, the hydrophilicity of the EBM samples results in a general spreading of the MC3T3-E1 cells all over the surface and over the underlaying plastic well, suggesting that only a limited number of cells lies on the samples built at higher degrees (30° and 45°). Since a low cytotoxicity is demonstrated by Ti6Al4V samples, a building orientation in relation to the building plate is shown to reduce the number of residual cells on EBM surfaces at 24h, following the MC3T3-E1 seeding (Figure 6). In fact, the cell proliferation reaches a higher level at 96h, demonstrating the same metabolic activity trend of the cells on all the EBM surfaces. In contrast, the SLM surfaces may eventually facilitate cellular adhesion and consequently promote the mineralization and osseointegration of the implant due to the higher attachment of the cellular suspension, as shown by the immunofluorescence staining (Figure 7). The SLM parts analyzed exhibit a different surface chemistry in relation to the EBM parts, and this may contribute to changes in cellular behavior as already reported [70]. The metabolic activity level of the cells on the SLM samples is generally higher and more uniform if compared with the EBM samples, as demonstrated by the roughness data related to the SLM surfaces. It has to be reported that the higher contact angles of the SLM surfaces are promoting, in this case, the cell attachment and consequently the proliferation of the cells. It has to be highlighted that previous works reported high but still hydrophilic contact angles [71]. Notably, both the EBM and SLM samples are promoting the mineralization of the MC3T3-E1 cells, as shown by the comparison with the plastic control levels of Ocn and Opn. It is known in the literature [63] that, during the intramembranous ossification in the crania of 16-day-old mouse fetuses, osteoblasts start to express *Bsp*, followed by the expressions of *Opn* and *Ocn*. *Ocn* mRNA is rarely expressed at the beginning stages of osteoblast differentiation, while it is detected in bone matrix after mineralization has significantly progressed. The upregulation of *Ocn*, in conjunction with the downregulation of *Opn*, observed on scaffold mineralization reflects a temporal variation in the gene expression pattern of osteoblasts wherein the *Opn* levels are lowered during *Ocn* expression [72]. It should be noted that Ocn is a bone-specific protein that is often considered as a late-stage marker of osteoblastic differentiation, typical of mature osteoblasts, whereas Opn is an extracellular matrix protein commonly used as early markers of osteogenic differentiation.

Hence, an early expression of *Ocn* can be indicative of the strong stimulatory effect of titanium scaffolds’ growth on bone cell differentiation [73,74]. Remarkably, the SLM samples are accelerating the mineralization process of the MC3T3-E1 cells, as demonstrated by the high level of *Ocn* at 15 and 30 days in relation to lower levels of *Opn* at the same time points. Moreover, the *Ocn* levels monitored on the SLM samples are significantly higher when compared with the EBM equivalents, demonstrating that promoting the initial attachment and arrangement of the cells can be crucial for differentiation and mineralization (Figure 9). It is worth noting that, at lower building degrees (0°, 15°), the *Ocn* levels on SLM samples are remarkably high, which indicates that the mineralization can be accelerated in the short term (15 days) by the building angle of the additively manufactured specimens (Figure 9). Probably, this trend can be explained by the higher number of cells observed by the metabolic activity data at 96h. Furthermore, the *Ocn* levels show similar trends in the long term (30 days) with a related similar reduction of the *Opn* values, strengthening the hypothesis that the mineralization can be influenced in the first days of differentiation (Figure 9). A proper comparison between the technologies used for the 3D printing of implants designed to favor cell adhesion and mineralization is still challenging. Furthermore, the osteoblast viability is mainly sustained by the chemical and physical properties of the surfaces, although the recent literature on the effectiveness of surface topography to modulate osseointegration is variable. Researchers should focus on different design features fabricated with distinct techniques to fully understand these correlations.

## 5. Conclusions

This work reports the analysis of additive manufacturing parameters on the osseointegration outcomes of Ti6Al4V parts. The design of a part that can be produced by PBF techniques can be optimized to tailor the surface area, the mechanical properties, and the porosity of the implant to facilitate and promote osseointegration and vascularization [19,21,23]. One of the main disadvantages of these technologies lies in the discontinuous powder melting process during the build, leading to uncontrollable and partially melted particles on the surface that minimize the surface area of the implants. For these reasons, several research studies have focused on the modification of the building angle during PBF processes to tailor and control the surface properties of the produced metal parts. Here, it was found that such a design feature has a limited influence on the cell attachment and differentiation trends due to the initial similar surface topography resulting from different building angles. The important outcome of this research relies on the results of the metabolic activity and differentiation of osteoblasts, which highlight a significant difference between the samples produced by selective laser melting and electron beam melting. Precisely, it was found that the SLM samples can promote the proliferation of preosteoblast cells and accelerate the mineralization significantly more than the EBM samples despite the fact that the EBM surfaces demonstrate a higher degree of mineralization of the cells in relation to the plastic controls. Moreover, the orientation of the samples to the build plate seems to have a limited effect on the osteogenic gene expression of the cells. The short-term osseointegration of the SLM specimens built at 0° or 15° may be accelerated in relation to the same gene expression on the samples with an orientation of 30° and 45°. This aspect has to be further analyzed and validated, but it can be considered an advantage regarding the osseointegration of complex parts within a bespoke implant especially for replacing missing tissues after massive removal surgeries. Additionally, the mineralization can be beneficial for the majority of joint arthroplasty, whereas it may be a disadvantage for components designed to be removed in the short term. Another important aspect that will be considered in future studies is the analysis of the outcomes of this research on bacterial adhesion, as already noted by the authors [75].

It can be concluded that the manufacturing technique has a substantial impact on the properties of 3D printed implants in comparison with the design approach used in the production.

## Figures and Tables

**Table 1:** Compositions and properties of the analyzed Electron Beam Melting (EBM) and (Selective Laser Melting (SLM) Ti64 powders. Data were collected following the related ASTM international standards.

Powder Property	EBM	SLM
Particle size analysis ^1^ (μm) d10 d50 d90	50 68 96	27.79 38.18 54.45
Powder density (g/cm^3^) ^2^ Apparent density	2.57	2.31
Chemical composition (wt %) ^3^ Al V O Fe Ti	6.42 3.88 0.13 0.18 Bal.	5.92 4.04 0.13 0.20 Bal.

^1^ ASTM B214 and B215 [60]. ^2^ ASTM B212 [61]. ^3^ ASTM F2924 and F1472 [62]. Bal., balance.

**Table 2:** Primer sequences used for quantitative real-time polymerase chain reaction.

Gene	Forward	Reverse
*BSP*	TTTATCCTCCTCTGAAACGGT	GTTTGAAGTCTCCTCTTCCTCC
*OCN*	CCGGGAGCAGTGTGAGCTTA	TAGATGCGTTTGTAGGCGGTC
*OPN*	GATGAACAGTATCCTGATGCC	TTGGAATGCTCAAGTCTGTG
*MEPE*	GTCTGTTGGACTGCTCCTCTT	CACCGTGGGATCAGGATACA
*GAPDH*	AGGTCGGTGTGAACGGATTTG	TGTAGACCATGTAGTTGAGGTCA

All quantitative data are presented as the mean ± SD, each experiment was performed three times, and, only for the qualitative experiments, the results from one representative experiment are shown. Significant differences were analyzed between the positive control (cells on plastic dish) and each type of scaffold using unpaired t-test. A value of *p* < 0.05 was considered statistically significant.

**Table 3:** Grouping information using the Tukey method and 95% confidence interval on the CA measurements of the SLM samples.

Angle SLM	N	Mean	Grouping *
0°	3	133.4	A
30°	3	122.6	A·····B
15°	3	119.1	·······B
45°	3	113.7	·······B

* Means that do not share a letter are significantly different.

**Table 4:** Grouping information using the Tukey method and 95% confidence interval on the CA measurements of the EBM samples.

Angle EBM	N	Mean	Grouping *
15°	3	111.3	A·
30°	3	104.1	A·······B
0°	3	97.8	·········B·······C
45°	3	91.2	·················C

* Means that do not share a letter are significantly different.

**Table 5:** Grouping information using the Tukey method and 95% confidence interval on the Ra measurements of the SLM samples.

Angle SLM	N	Mean	Grouping *
30°	3	24.4	A·
0°	3	21.9	A·······B
15°	3	20.8	A·······B
45°	3	17.4	·········B

* Means that do not share a letter are significantly different.

**Table 6:** Grouping information using the Tukey method and 95% confidence interval on the *Ra* measurements of the EBM samples.

Angle EBM	N	Mean	Grouping *
15°	3	51.0	A
0°	3	44.7	A
30°	3	27.8	B
45°	3	25.6	B

* Means that do not share a letter are significantly different.
